# The Association between the Occurrence of Asthma and Antecedents of Exposure to Environmental Tobacco Smoke in the Previous Year in Children: An Incidence-Density Study

**DOI:** 10.3390/ijerph19052888

**Published:** 2022-03-02

**Authors:** Hayat Bentouhami, Lidia Casas, Joost Weyler

**Affiliations:** 1 Social Epidemiology and Health Policy (SEHPO), University of Antwerp, 2610 Wilrijk, Belgium; lidia.casas@uantwerpen.be (L.C.); joost.weyler@uantwerpen.be (J.W.); 2 StatUa Statistics Centre, University of Antwerp, 2000 Antwerpen, Belgium

**Keywords:** asthma, environmental tobacco smoke, maternal smoking, paternal smoking, incidence-density study

## Abstract

In previous studies, the strength of the association between childhood asthma and environmental tobacco smoke (ETS) differed depending on the way ETS was assessed and the type of study conducted. We investigated the relationship between asthma occurrence in children and recent exposure to ETS based on an incidence-density study driven by the explicit formulation of a theoretical design. Additionally, we assessed whether the relationship is modified by perinatal ETS exposure and parental inhalation atopy. The event was conceptualized as ‘first doctor’s diagnosis of asthma’. Population time was probed by sampling population moments. Exposure to ETS was conceptualized as recent exposure (1 year prior to diagnosis or at sampling) and perinatal exposure (in utero and/or during the first year of life). Thirty-nine events and 117 population moments were included. There was no indication for effect modification by perinatal exposure to ETS or parental inhalation atopy. After adjustment for confounding, an association was observed between occurrence of a first asthma diagnosis and recent ETS exposure: incidence-density ratio 4.94 (95% confidence interval 1.21, 20.13). Asthma occurrence in children is associated with recent exposure to ETS, and this association seems not to be modified by perinatal ETS exposure or parental inhalation atopy.

## 1. Introduction

According to the Global Burden of Disease (GBD) study, asthma affected approximately 262 million people and caused 461,000 deaths in 2019 [1,2]. Among children, asthma is the most common chronic disease [1]. Asthma is a heterogeneous disease which is (usually) characterized by chronic airway inflammation and defined by a history of respiratory symptoms (wheezing, shortness of breath, chest tightness and cough). These symptoms can vary over time and in intensity, and occur together with variable expiratory airflow limitation [3]. The underlying causal mechanisms in the development of asthma are still not completely understood. However, there is general consensus among experts that the inception and persistence of asthma is influenced by gene–environment interactions and that a ‘window of opportunity’ exists during the perinatal period (i.e., in utero and during the first year of life) when the immune system is still developing and environmental risk factors have the opportunity to influence this development, and as a consequence may influence the onset of asthma [3].

Based on insights from in vitro studies and observational studies, the current advice for the prevention of asthma is to avoid exposure to ETS during pregnancy and in the first year of life [3]. In a systematic review and meta-analysis of cohort studies investigating the effect of smoking by parents or household members on the risk of asthma, Burke et al. reported that in young children (aged ≤2 years) the strongest effect was observed for in utero exposure to ETS (through active smoking of the mother). This effect weakened with increasing age, but remained present. Postnatal exposure to ETS, on the other hand, was only associated with the incidence of asthma in older children (aged 3–18 years) [4]. In a recent systematic review and meta-analysis by He et al., it was shown that exposure to ETS (for any exposure time window) was associated with a higher risk of a doctor’s diagnosis of asthma in children [5]. However, differences in the strength of the associations were observed depending on the way ETS was conceptualized (in utero exposure vs. postnatal exposure). In utero ETS exposure was more strongly associated with asthma occurrence than postnatal ETS exposure [5]. In a subgroup analysis, He et al. divided the studies included according to type (case–control studies/cross-sectional studies vs. cohort studies). In case–control studies and cross-sectional studies, in utero ETS exposure was more strongly associated with asthma occurrence than postnatal ETS exposure, while in cohort studies this was the other way around [5].

Several biological mechanisms can explain the relationship between exposure to ETS and the occurrence of asthma. Two indirect effects are the modification of the development of the immune system by suppressing the Th1 immune response and enhancing the Th2 immune response, and the modulation of the ubiquitin–proteasome pathway [6,7]. The toxicity of the chemicals in tobacco smoke can also directly affect the respiratory epithelium and smooth muscle tissues of the lungs [6,7]. Even though the relationship between exposure to ETS and the occurrence of asthma in children has been investigated extensively, only a few studies operationalized exposure to ETS in several ways (both in utero exposure and postnatal exposure) [8,9,10,11]. However, and to the best of our knowledge, none of these studies investigated the relationship between a doctor’s diagnosis of asthma with recent exposure to ETS (outside the perinatal period but prior to asthma onset), even though it is hypothesized that ETS exposure can also have direct effects on the lungs. Therefore, the aim of this study is to investigate the relationship between current asthma occurrence in children and antecedents of recent ETS exposure by conducting an incidence-density study. Additionally, we assessed whether the relationship between current asthma occurrence in children and recent ETS exposure is modified by perinatal exposure to ETS and parental inhalation atopy.

## 2. Materials and Methods

### 2.1. Research Question and Theoretical Design

The aim of the study led to the following research question: What is the relationship between asthma occurrence in children and antecedents of recent exposure to ETS (1 year prior to diagnosis), and is this relationship modified by perinatal exposure to ETS and parental inhalation atopy?

In order to be able to answer the research question, a theoretical design was formulated: current incidence (density) of asthma in children as a function of prior exposure (1 year prior) to ETS taking into account effect modification by perinatal exposure to ETS and parental inhalation atopy and adjusting for confounding by age, sex, parental education and daycare attendance, and in case of no indication for effect modification also for perinatal exposure to ETS and parental inhalation atopy.

The domain is children prior to puberty.

A directed acyclic graph (DAG) was constructed to determine what covariates to adjust for.

### 2.2. Design of Data Collection

Data were collected in the Prospective data collection project on the Influence of Perinatal factors on the Occurrence of Asthma and Allergies (PIPO) [12,13]. This project started in 1997 in the province of Antwerp, including 1128 children and collecting information on the mothers (pregnancy) and offspring in order to investigate the perinatal risk factors for the occurrence of asthma and other allergic illnesses in childhood. Information on outcome, exposure and relevant characteristics was obtained from the mother’s and father’s questionnaires, the questionnaire at the second home visit, the bi-annual questionnaires between birth and 4 years of age and the annual questionnaires between 4 and 8 years of age.

We defined current incidence density as the number of events (cfr. infra) divided by the summation of all observation periods ‘at risk’ for the event in an observed population, for an observation period going to zero (instantaneous incidence density). It is clear, however, that the assessment of instantaneous incidence density is not feasible, as the measurement of exposed and unexposed population time becomes unfeasible when the observation period actually goes to zero. Therefore, a quasi-incidence density sampling over a non-zero observation period was set up [14].

#### 2.2.1. Sampling

Quasi-incidence density of asthma was assessed in an observation period of 7 years (between 1 and 8 years of age) in order to cover the domain and to collect a sufficient number of events.

##### Events

Events were conceptualized as parent-reported first doctor’s diagnoses of asthma. A parent-reported first doctor’s diagnosis of asthma was defined as answering for the first time ‘yes’ to the question “Was your child suffering from asthma in the previous six months and was this confirmed by a doctor?” (between 1 and 4 years) or “Was your child suffering from asthma in the previous 12 months and was this confirmed by a doctor?” (between 4 and 8 years of age). Events were therefore first doctor’s diagnoses of asthma between the age of 1 and 8 years. Events under the age of 1 year were excluded from the study.

##### Population Time

As measuring the entire population time in a dynamic exposure experience is almost impossible and not essential for the valid estimation of the incidence-density ratio [14], we decided to probe population time in two stages. First, all records (at each follow-up) from the PIPO project leading to the collection of information were considered as a probe for the evolving population time in the participating children in the project. We refer to these as population moments. At each follow-up within the PIPO project, all population moments still ‘at risk’ for developing the event were entered in a ‘risk set’. This ‘risk set’ contained all population moments still ‘at risk’ for the event within the same observation period as the period for collecting the events (between 1 and 8 years of age). Secondly, from this ‘risk set’, a random and unmatched sample of population moments was taken as a probe of the study base, and after merging with the events, extensive information on the sampled records (events and population moments) was retrieved.

#### 2.2.2. Recent Exposure to Environmental Tobacco Smoke

Recent exposure to ETS was defined as exposure to ETS 1 year prior to the first doctor’s diagnosis of asthma or sampling of the population moment. This information was retrieved from the bi-annual and annual questionnaires in which the parents were asked the following question: In the last 6 months/12 months, was your child regularly exposed to tobacco smoke (cigarettes, cigars, pipe)? (‘Regularly’ is most days of the week.)

All exposure variables were dichotomized into ‘exposed’ vs. ‘unexposed’.

#### 2.2.3. Relevant Characteristics

**Figure 1:**
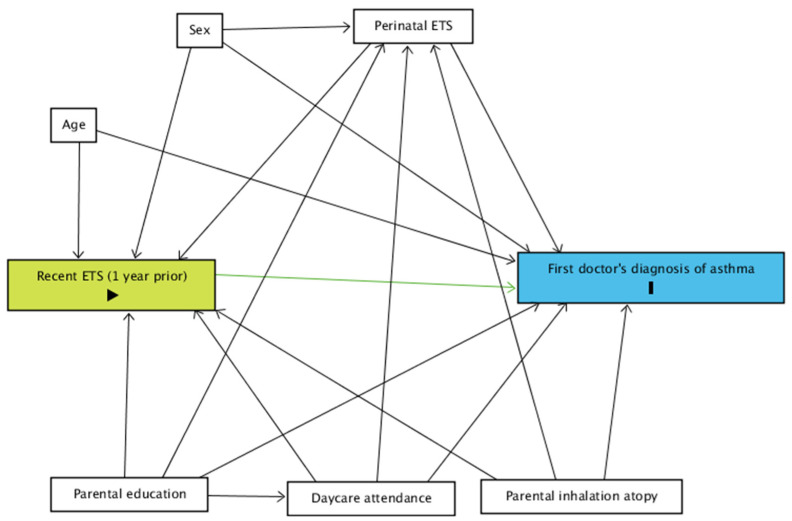
DAG for the relationship between the current occurrence of a first doctor’s diagnosis of asthma and recent exposure to ETS. Green: exposure; blue: event; ETS: environmental tobacco smoke.

A directed acyclic graph (DAG) was constructed using ‘DAGitty v.3.0’ for the relationship between recent exposure to ETS and asthma diagnosis (Figure 1) [15].

Perinatal exposure to ETS (in utero exposure of the child to ETS and/or exposure to ETS during the first year of life, yes vs. no) and parental inhalation atopy (self-reported hay fever, yes vs. no) were considered as potential modifiers.

Age (in months), sex (male vs. female), parental education (high vs. low) and ever attending daycare were considered as potential confounders. In case of no indication for effect modification, perinatal exposure to ETS and parental inhalation atopy were considered as potential confounders as well.

### 2.3. Design of Data Processing

#### 2.3.1. Handling of Missing Data

Missing data were imputed by applying Multiple Imputation by Chained Equations (MICE) [16]. In the imputation model, all variables of interest for the model (cfr. supra) were included. The number of imputed datasets was set at 30 and the logistic regression method was used for imputation.

#### 2.3.2. Statistical Modelling

Data were summarized in a 2 × 2 table and odds ratios (ORs) were calculated for the estimation of the crude (quasi-) incidence-density ratios (IDRs) with estimation of 95% confidence intervals (CI). As the IDRs were estimated by calculating ORs, multiple logistic regression was applied to assess the presence of effect modification and to control for confounding. An α-level of 0.20 was used to decide on the inclusion of an interaction term.

In order to facilitate the interpretation of the regression models, all relevant statistics are presented (regression coefficients, standard errors, IDRs and 95% CIs) [17]. All statistical procedures were performed in R version 1.4.1106 [18].

### 2.4. Ethics Approval

The medical ethics committee of the University Hospital of Antwerp granted approval for all parts of the project (UA A06 10) and informed written consent was obtained from the parents for each assessment during the project.

## 3. Results

### 3.1. Sampling

In total, 39 events (first doctor’s diagnoses of asthma between 1 and 8 years of age) were identified. A sample of 117 unmatched population moments was randomly taken from the ‘risk set’. In total, the number of records was 156.

### 3.2. Relevant Characteristics

Table 1 shows the characteristics of the events (*n* = 39) and the population moments sampled as a probe of population time (*n* = 117). In the events, the proportion of females was smaller compared to the population moments. Events were more often exposed to ETS in the year prior to diagnosis and perinatally compared to the population moments. A higher proportion of events had parents with inhalation atopy compared to the population moments.

### 3.3. Missing Data

There were no missing data for the outcome (first doctor’s diagnosis of asthma), age and sex. In total, 27 (17.3%) records had at least one missing value for the remaining characteristics (recent ETS exposure, perinatal ETS exposure, parental education, daycare attendance and parental inhalation atopy).

### 3.4. Relationship between Current Occurrence of First Doctor’s Diagnosis of Asthma and Recent Exposure to ETS

#### 3.4.1. Crude Incidence-Density Ratios

In the crude analysis, an association was observed between the occurrence (incidence density) of a first doctor’s diagnosis of asthma and recent exposure to ETS: IDR 2.62 (95% CI 0.92, 7.52).

#### 3.4.2. Effect Modification by Perinatal ETS Exposure and Parental Inhalation Atopy

We were not able to assess effect modification by parental inhalation atopy because of empty cells. We found no evidence for effect modification by perinatal exposure to ETS because the estimation of the regression coefficient of the interaction term exceeded the α-level of 0.20 (Table 2).

#### 3.4.3. Adjustment for Confounding

After adjustment for confounding by age, sex, parental education, perinatal exposure to ETS, daycare attendance and parental inhalation atopy (Table 3), the association observed between the occurrence of a first doctor’s diagnosis of asthma and recent exposure to ETS was: IDR 4.94 (95% CI 1.21, 20.13).

## 4. Discussion

In this study, we observed a strong (IDR 4.94 [95% CI 1.21, 20.13]) association between the occurrence of a first doctor’s diagnosis of asthma and recent exposure to ETS (1 year prior to diagnosis). These findings imply that exposure in early childhood, also outside of the perinatal window, is associated with the occurrence of asthma. Other studies mainly focused on in utero exposure to ETS and/or exposure in early life (future occurrence as a function of current exposure instead of current occurrence as a function of past exposure) [10,19].

Additionally, we evaluated whether the association was modified by perinatal exposure to ETS. We did not find an indication for effect modification by perinatal ETS exposure.

Even though we observed a strong and statistically significant association (α-level 0.05) between the occurrence of a first doctor’s diagnosis of asthma and recent exposure to ETS, the sample size of our study is rather small. We were only able to identify 39 first doctor’s diagnoses of asthma. Therefore, we are aware that the results of this study should be interpreted with caution.

Some studies assessed ETS exposure and the occurrence of asthma cross-sectionally and found an association between ETS exposure and asthma occurrence [20,21]. However, in cross-sectional studies the strength of the association might be underestimated due to misclassification of the exposure. Parents of children experiencing asthmatic symptoms might stop exposure of their child to ETS early on. At the same time, recall bias and social desirability bias might lead to misclassification of the exposure in cross-sectional studies. Our study is (to the best of our knowledge) the first in which exposure to ETS is reconstructed as an antecedent 1 year prior to asthma diagnosis. Our findings are supported by previous findings from studies investigating the effects of exposure to ETS on markers of inflammation on a molecular biological level.

Our findings confirm the hypothesis proposed in studies on a molecular biological level assessing the direct effects of ETS on pathways of inflammation: that is, apart from being involved in the development of the immune system, ETS can also have direct inflammatory effects and act as a trigger [22].

In several studies, it was observed that healthy children exposed to ETS had higher levels of serum IL-4, IL-5, total IgE and a higher absolute number of blood eosinophils and also elevated levels of TNF-α and INF-γ [23,24,25]. In another study, elevated IL-5, IL-6, TNF-α and INF-γ after 1 h were also observed in healthy adult non-smokers that were exposed to tobacco smoke [26].

Asthma is a complex disease, and diagnosing it, especially in young children, is challenging [3]. In our study, we only included first doctor’s diagnoses of asthma, but we are aware that this could have led to the missing of events. However, we preferred this type of misclassification as defining the event based on parent-reported symptoms would have inflated the number of events by observations that are probably not related to ETS (but to infections). This would have led to an underestimation of the true effect of ETS. For the same reason, we did not include events under the age of 1 year, because in these cases, wheezing and other respiratory symptoms occur very frequently (e.g., as a consequence of mild viral respiratory tract infections), making a diagnosis of asthma even more difficult [27].

The main exposure (recent exposure to ETS) was assessed 1 year prior to diagnosis of asthma. We did not take into account the duration of the exposure or the dose, although Chau-Etchepare et al. pointed out that this might be important to consider [22]. We did not consider the duration of exposure because we would in some cases not be able to distinguish between perinatal exposure and recent exposure. We advise that larger studies should take the duration of exposure into account.

One of the strengths of our study is that the research question was translated into a theoretical design and the corresponding method of data collection and method of data processing was chosen [14,28,29,30]. We performed an incidence-density study allowing us to sample events and population moments and to reconstruct the antecedents carefully (exposure and other relevant characteristics) before the occurrence of the event or at sampling as a population moment. In order to achieve this, we made use of data from the PIPO project, where extensive information about the pregnancy of the mothers and information on the offspring was collected repeatedly from birth up until the age of 8 years. This extensive information was collected prior to occurrence of the event in the PIPO prospective data collection project, including the information on exposure to ETS. This limits misclassification for the exposure (recall bias, etc.). The extensive information collected at regular time-points in the PIPO project allowed us to take into account relevant exposures and characteristics over the whole life span of the child (modifiers and confounders) at relevant time-points (prior to the diagnosis of asthma). Lastly, we dealt with missing data by using multiple imputation by chained equations for imputation. Performing the modelling on complete cases only would have led to biased results [16].

## 5. Conclusions

In conclusion, we found a strong association between the occurrence of asthma and recent exposure to ETS. This association seems not to be modified by perinatal exposure to ETS. We advise further studies to assess the relationship between asthma and exposure to ETS in more detail (e.g., dose and duration of exposure) with larger sample sizes and with a similar design (theoretical design, design of data collection and design of data processing carefully considering temporal aspects between exposure(s) and outcome).

## Figures and Tables

**Table 1:** Characteristics of the children in whom the events (first doctor’s diagnoses of asthma, *n* = 39) were sampled from and population time was probed from (population moments, *n* = 117).

	Events (*n* = 39)	Population Moments (*n* = 117)
Age, months, median (IQR)	48 (30)	42 (42)
Sex, female, *n* (%)	10 (25.6)	65 (55.6)
Parental education, high, *n* (%)	31 (86.1)	103 (90.4)
Daycare attendance, yes, *n* (%)	27 (79.4)	95 (84.1)
Parental inhalation atopy, yes, *n* (%)	25 (71.4)	53 (46.1)
Perinatal ETS, yes, *n* (%)	17 (48.6)	29 (25.9)
Recent ETS exposure, yes, *n* (%)	6 (15.4)	8 (6.8)

IQR: interquartile range; ETS: environmental tobacco smoke.

**Table 2:** Evaluation of effect modification of the relationship between the occurrence of a first doctor’s diagnosis of asthma and recent exposure to ETS by perinatal exposure to ETS.

	β	SE	IDR	95% CI
Recent exposure to ETS for perinatal exposure to ETS = unexposed	0.78	1.25	2.18	0.19–25.31
Recent exposure to ETS for perinatal exposure to ETS = exposed	0.42	0.65	1.52	0.42–5.49
Interaction term	−0.36	1.42 ^§^	-	-

ETS: environmental tobacco smoke; ^§^: *p* > 0.20.

**Table 3:** Crude and adjusted associations for the relationship between the occurrence of a first doctor’s diagnosis of asthma and recent exposure to ETS.

	β	SE	IDR	95% CI
Recent exposure to ETS (crude)	0.96	0.54	2.62	(0.92, 7.52)
Recent exposure to ETS (adjusted) *	1.60	0.72	4.94 ^†^	(1.21, 20.13)
Constant	−2.12	0.98	0.12 ^†^	(0.02, 0.81)
Age	0.01	0.01	1.01	(0.99, 1.02)
Sex	−1.48	0.49	0.23 ^†^	(0.09, 0.59)
Parental education	0.38	0.78	1.47	(0.32, 6.82)
Daycare attendance	−0.46	0.57	0.63	(0.21, 1.95)
Perinatal exposure to ETS	0.80	0.48	2.22	(0.87, 5.67)
Parental inhalation atopy	1.16	0.48	3.18 ^†^	(1.23, 8.20)

ETS: environmental tobacco smoke; * Adjusted for confounding by age, sex, parental education, daycare attendance, perinatal exposure to ETS and parental inhalation atopy; ^†^ *p* < 0.05.

## Data Availability

The data that support the findings of this study are available on request from the corresponding author. The data are not publicly available due to privacy or ethical restrictions.
